# Angiopoietin-like protein 2 expression is suppressed by angiotensin II via the angiotensin II type 1 receptor in rat cardiomyocytes

**DOI:** 10.3892/mmr.2020.10919

**Published:** 2020-01-09

**Authors:** Shuya Wang, Ying Li, Wei Miao, Hong Zhao, Feng Zhang, Nan Liu, Guohai Su, Xiaojun Cai

Mol Med Rep 14: 2607-2613, 2016; DOI: 10.3892/mmr.2016.5544

Following the publication of this article, an interested reader drew to our attention that, in the Materials and methods section of the paper, on p. 2608 in the *Immunofluorescence* subsection, the authors had included a catalog number (cat no. 6487) that belonged to α-actinin, not α-smooth muscle actin (α-SMA).

After having investigated this matter with the authors, they were able to confirm that, although both α-SMA and α-actinin antibodies were employed in this study to investigate purification of the cardiomyocytes, the catalog number for α-SMA should have been referenced as ab5694 from Abcam, and the catalog number for α-actinin from Cell Signaling Technology is #6487. The authors were also able to confirm that staining with α-SMA was presented in Fig. 1 of their article, and therefore the relevant catalogue number should have been listed on p. 2608 as ab5694 (Abcam) for α-SMA, not #6487.

The authors express their gratitude towards the reader who identified this error, and regret any inconvenience that this mistake has caused.

